# Real-World Evaluation of PCI Guidance Using Dynamic Coronary Roadmap: A DCR4Contrast Trial Secondary Analysis

**DOI:** 10.1016/j.jscai.2024.102504

**Published:** 2025-02-07

**Authors:** Breda Hennessey, Haim Danenberg, Frédéric De Vroey, Ajay J. Kirtane, Manish Parikh, Dimitrios Karmpaliotis, Aaron Strobel, Alejandro Curcio, Martijn S. van Mourik, Peter Eshuis, Javier Escaned, John C. Messenger

**Affiliations:** a Interventional Cardiology Section, Hospital Clinico San Carlos (Instituto de Investigación Sanitaria del Hospital Clínico San Carlos, IdISSC), Complutense University of Madrid and Centro de Investigación Biomédica en Red en Enfermedades Cardiovasculares (CIBERCV), Madrid, Spain; b Department of Cardiology, Blackrock Clinic, Dublin, Ireland; c Interventional Cardiology, Heart Institute, Hadassah–Hebrew University Medical Center, Jerusalem, Israel; d Interventional Cardiology Division, Wolfson Medical Center Holon, Holon, Israel; e Department of Cardiology, Grand Hôpital de Charleroi, Charleroi, Belgium; f Division of Cardiology, NewYork-Presbyterian Hospital/Columbia University Irving Medical Center, New York, New York; g Cardiovascular Research Foundation, New York, New York; h Division of Cardiology, NewYork-Presbyterian/Brooklyn Methodist Hospital, New York, New York; i Division of Cardiology, Morristown Medical Center, Morristown, New Jersey; j Division of Cardiology, Department of Medicine, University of Colorado School of Medicine, Anschutz Medical Campus, Aurora, Colorado; k Division of Cardiology, Baptist Health Heart Institute, Little Rock, Arizona; l Department of Cardiology, Hospital de Fuenlabrada, Madrid, Spain; m Image Guided Therapy Systems, Philips Medical Systems, Best, the Netherlands

**Keywords:** contrast-induced acute kidney injury, Dynamic Coronary Roadmap imaging-guided technique, iodinated contrast reduction, percutaneous coronary intervention, real-time overlay

## Abstract

**Background:**

Iodinated contrast used during percutaneous coronary intervention (PCI) risks contrast-induced acute kidney injury (CI-AKI). Reducing this risk is essential as PCI procedures become more complex. Dynamic Coronary Roadmap (DCR) is a PCI tool that overlays a virtual roadmap on fluoroscopy and has been shown to reduce contrast use.

**Methods:**

This secondary analysis from the Dynamic Coronary Roadmap for Contrast Reduction (DCR4Contrast) study evaluates the feasibility of obtaining high-quality roadmaps suitable for PCI, its influence on contrast reduction and the relationship between PCI complexity and roadmap quality, and its effect on the contrast-sparing capabilities of DCR compared with standard angiographic guidance. The study was prospective and randomized, conducted in 6 centers across Europe (n = 3), Israel (n = 1), and the United States (n = 2). Patients were assigned to either DCR guidance or conventional guidance, and contrast usage and roadmap quality were compared.

**Results:**

The study included 365 patients (181 DCR and 184 control). Both groups were comparable in demographics and procedure characteristics. The DCR arm showed clinically usable roadmap quality in 97.2% of cases. Contrast volume was significantly lower with DCR guidance and lowest when the roadmap scored better: 63.5 ± 50.3 mL for “DCR good” (n = 147) vs 79.3 ± 42.8 mL for “DCR fair/poor” (n = 34) vs 90.2 ± 53.3 mL for “Control” (n = 184) (*P* < .001). DCR’s efficacy increased with PCI complexity (using the Synergy Between Percutaneous Coronary Intervention with Taxus and Cardiac Surgery [SYNTAX]) score of the treated vessel [SSv] as an index): 71.4% of first tertile (SSv < 4), 79.7% of second tertile (4 ≤ SSv < 8), and 93.1% of third tertile (SSv ≥ 8) scored “very good” or “good” (*P* < .05).

**Conclusions:**

This multicenter study shows that DCR technology provides consistent high-quality roadmap support, reducing iodinated contrast usage significantly, particularly as PCI complexity increases.

## Introduction

Percutaneous coronary intervention (PCI) is a widely used therapeutic method for the treatment of coronary artery disease. However, administering iodinated contrast during PCI can lead to contrast-induced acute kidney injury (CI-AKI), which has consistently been shown to be associated with poorer clinical outcomes and increased costs, particularly in patients with pre-existing renal dysfunction or diabetes mellitus.^1^, ^2^, ^3^, ^4^, ^5^, ^6^, ^7^, ^8^ There is an increasing proportion of patients who are at risk of developing CI-AKI due to their comorbidities, or we are required to intervene on complex coronary or multivessel disease, which typically requires significant contrast volumes.^9^

**Figure 1:**
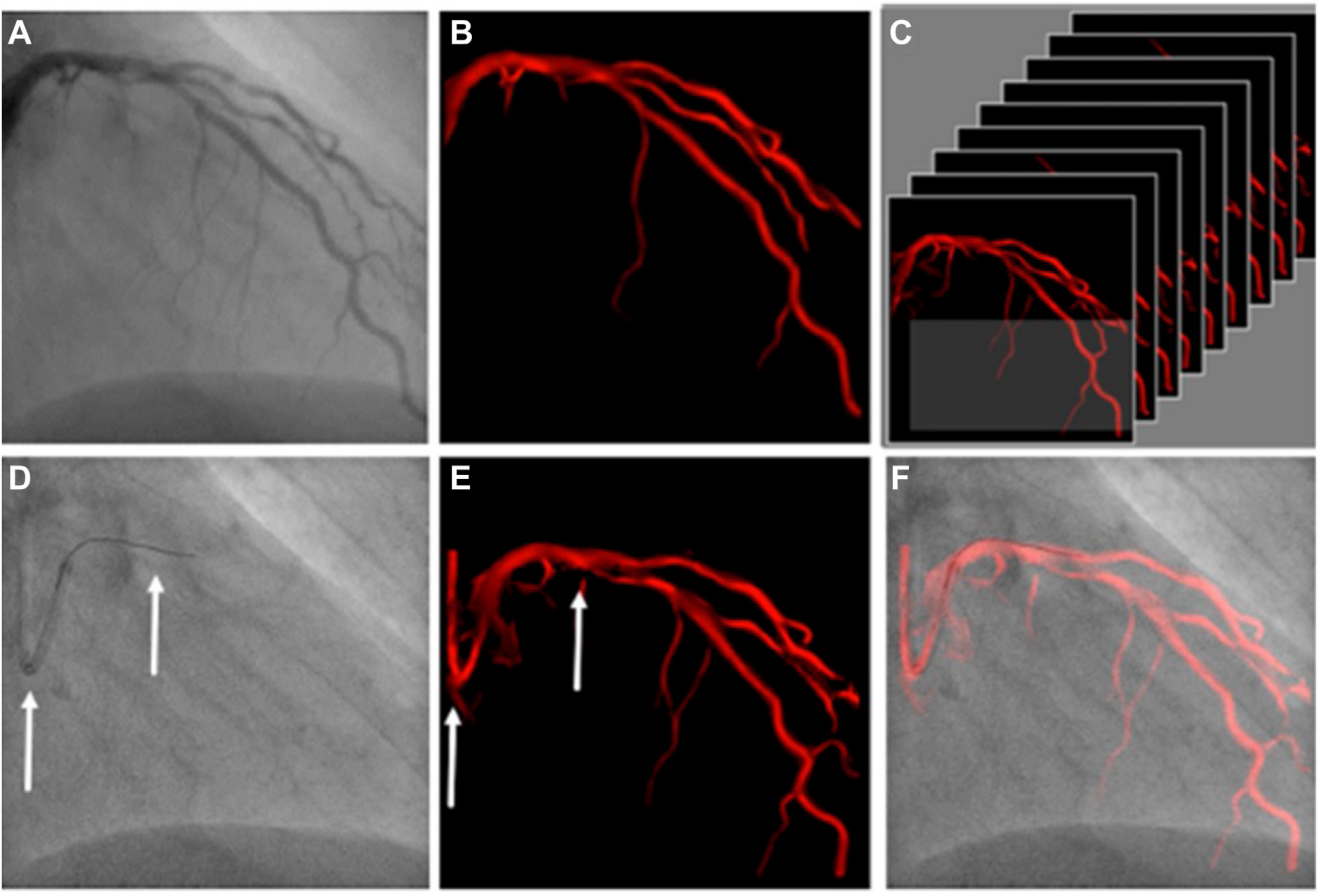
**Philips Dynamic Coronary Roadmap navigational tool for vessel wiring and stent placement.** (**A**) A coronary angiogram is automatically analyzed on contrast density and (**B**) converted into a mask for every angiographic image. (**C**) A heart cycle of masks is stored in a library, each one defined by its C-arm position. (**D**) If a corresponding C-arm position is accomplished, the software analyzes the guide catheter and wire shape (fixed curvatures, see arrows) in the fluoroscopy image. (**E**) A search in the library for a mask with similar fixed curvatures (see arrows) is performed, and (**F**) the best matching mask is superimposed in red color on the fluoroscopy image in real-time using these fixed curvature features to produce the Dynamic Coronary Roadmap overlay to assist device navigation during percutaneous coronary intervention. Figure 1 is adapted from the study by Piayda et al.^11^ Copyright of the figure belongs to the original publisher, *European Journal of Medical Research*.

The use of Dynamic Coronary Roadmap (DCR; Philips Medical Systems) technology during PCI has been proven to reduce the amount of iodinated contrast used and therefore may decrease the risk of CI-AKI.^9^^,^^10^ The DCR system generates a real-time, automatic motion-compensated angiographic roadmap of the coronary arteries on fluoroscopy, which can facilitate the accurate positioning of guide wires, balloons, and stents during PCI (Figure 1). While the efficacy of DCR has been demonstrated in previous studies, roadmap quality and its effect on the contrast-sparing ability of DCR have not been evaluated in real-world settings with varying PCI complexities.^9^^,^^10^

In this subgroup analysis of the Dynamic Coronary Roadmap for Contrast Reduction (DCR4Contrast) Trial, we investigated the feasibility of obtaining coronary roadmaps with adequate quality for PCI guidance in a real-world context. We also investigated whether roadmap quality had an influence on contrast volume usage. Finally, the interaction between DCR use and PCI complexity in terms of contrast reduction was also studied. The results of this study may help physicians decide when and how to apply DCR technology in clinical practice and ultimately contribute to improved patient safety during PCI procedures.

## Methods

The rationale, design, and results of the DCR4Contrast Trial have recently been published.^9^^,^^10^ In brief, the DCR4Contrast Trial was a multicenter, international, prospective, unblinded, stratified 1:1 randomized controlled trial conducted in 6 tertiary referral centers in Europe (n = 3), Israel (n = 1), and the United States (n = 2). The study population included patients aged >18 years, with a signed consent form, who were undergoing ad hoc or planned nonemergent PCI. Patients were randomized to undergo PCI performed using DCR guidance (treatment group) or using conventional angiographic guidance (control group). Balanced 1:1 randomization was performed according to the type of procedure (ad hoc or planned) and the number of vessels planned to be treated.^9^^,^^10^

The primary objective of the main trial was to assess whether using DCR reduces the total iodinated contrast volume used for PCI procedures (primary end point) compared with the control group without DCR. The secondary objective was to assess the total number of contrast enhanced cine angiographic runs per PCI procedure (secondary end point) in the DCR and control arms.^9^^,^^10^ Patients who were randomized to the DCR group underwent PCI using the DCR technology to aid in the advancement of coronary wires, balloons, stents, and other PCI diagnostic or therapeutic equipment. Patients assigned to the standard angiographic guidance arm underwent PCI without DCR. In both arms, the operator was expected to follow their normal clinical practice for PCI and the treatment of coronary artery disease. A prespecified treated vessel Synergy Between Percutaneous Coronary Intervention with Taxus and Cardiac Surgery (SYNTAX) score (SSv) (ie, SSv refers to the treated vessel SYNTAX score) was calculated as a metric of anatomical vessel complexity for each vessel undergoing PCI.

In the DCR arm, the DCR system was used to generate real-time roadmaps of the coronary anatomy. The quality of these roadmaps was rated by the interventional cardiologist performing the procedure at the time of the intervention using a Likert scale of “very good,” “good,” “fair,” “poor,” or “very poor.” Roadmap quality definitions were prespecified (Table 1) and were evaluated based on how complete and accurate the coronary artery tree was represented and whether the location of the roadmap during fluoroscopy was correct (eg, compared with the location of the interventional guiding catheter, guide wire, or device). Scoring the overall quality and accuracy of the roadmap during PCI was performed directly after the procedure and left to the discretion of the investigator performing the PCI using Table 1 as a reference.^9^^,^^10^

**Table 1:** Roadmap quality score was determined based on evaluation of coronary artery tree representation and overlay location during fluoroscopy.

Roadmap quality score	Roadmap represents overall coronary artery tree correctly?		Was the roadmap overlay location correct during fluoroscopy the majority of the PCI? (eg, compared with location of interventional guiding catheter, guide wire, and device)
Very good	Yes, all coronary artery tree details are captured	AND	Yes, the overlay location is in the correct location throughout the PCI
Good	Yes, only some insignificant details of the artery tree are omitted	AND	Yes, the overlay location is in the correct location most of the PCI
Fair	Yes, although some coronary anatomy parts are omitted, the roadmap is still clinically usable	AND	Yes, although the overlay location is not always correct, it still provides sufficiently correct navigation guidance during the PCI
Poor	No, relevant coronary arteries are omitted from the roadmap	OR	No, the overlay location is not correct for most of the PCI and not usable for navigation guidance
Very poor	No, relevant coronary arteries are omitted from the roadmap, not providing an overall artery tree overview	OR	No, the overlay location is almost never correct during the PCI and not usable for navigation guidance

PCI, percutaneous coronary intervention.

The focus of this report is the prespecified secondary or subgroup analysis to analyze the primary and secondary end points (iodinated contrast volume usage and number of cineangiography runs during PCI, respectively) in the DCR arm for the safety population (all randomized subjects who underwent PCI, where the subjects are allocated in the DCR and Control group as treated) by overall roadmap quality and accuracy, and by PCI complexity using the SSv of the treated vessel as an index. ^9^^,^^10^

Statistical analyses were performed using SAS (SAS Institute) and/or R statistical software (https://www.r-project.org/). Standard statistical methods were used for summarization and analysis. Data are presented as mean ± SD with 95% CIs for continuous variables with a normal distribution, median (Q1-Q3) (with Q1 and Q3 being the first and third quartile, respectively) for continuous variables with a nonnormal distribution, and as frequency (percentage) for categorical variables. χ^2^ test or Fisher exact test was performed for categorical variables and analysis of variance or Kruskal–Wallis test for continuous variables. A 2-sided α of 5% was used for these analyses, and no log transformation was performed. In this study, we carried out hypothesis-generating tests and did not apply adjustments to the α level to control for type I error due to multiplicity. Our primary aim was to explore potential associations, making multiplicity adjustments unnecessary for this exploratory analysis.

The study was approved by the institutional review board or medical ethics committee of the participating hospitals. All enrolled eligible patients provided written informed consent. The trial was funded by Philips Medical Systems (Best, the Netherlands). This trial is registered at ClinicalTrials.gov (unique identifier: NCT04085614).

## Results

**Supplementary Figure S1:**
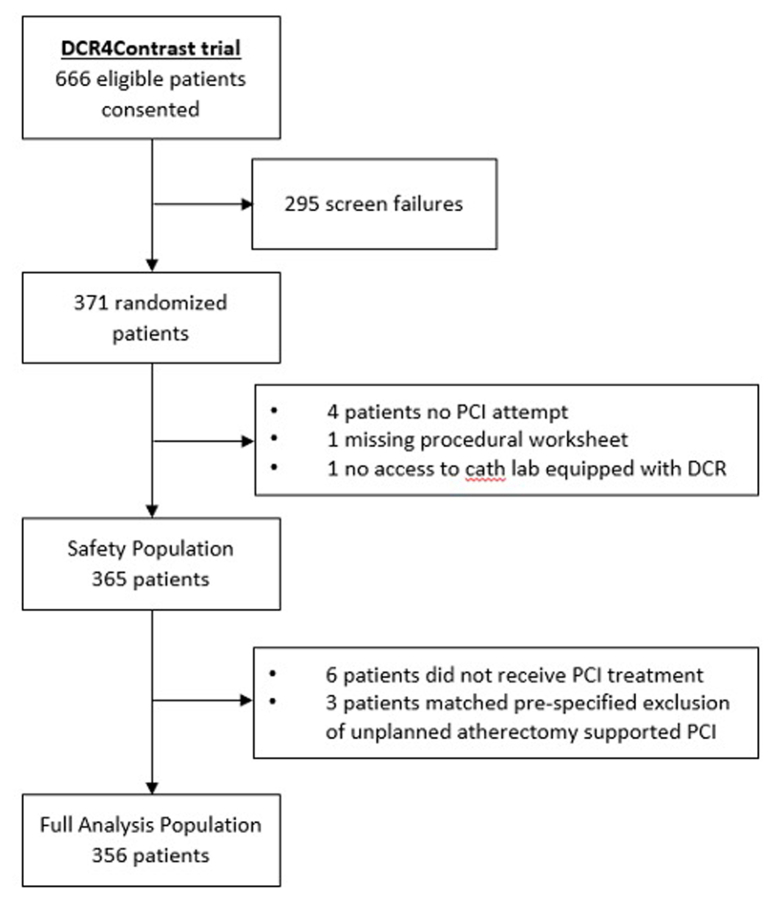
**Study flow of the DCR4Contrast randomized controlled trial. DCR,** Dynamic Coronary Roadmap; PCI, percutaneous coronary intervention.

Between November 2019 and February 2023, a total of 371 patients were enrolled and randomized in 6 centers. After exclusion of 6 patients in which no PCI was performed, the safety population consisted of 365 patients divided between the DCR-guided PCI group (181 patients) and the angiography-guided PCI group (184 patients).^9^^,^^10^ For the present analysis, we prespecified that the safety population would be used, as shown in Supplemental Figure S1. The safety population differed from the full analysis population in the following aspects: it included all randomized subjects who underwent any PCI attempt or another procedure such as coronary artery bypass graft surgery. It did not specify end point data availability, and it analyzed data based on the actual treatment subjects received, regardless of randomization.

**Figure 2:**
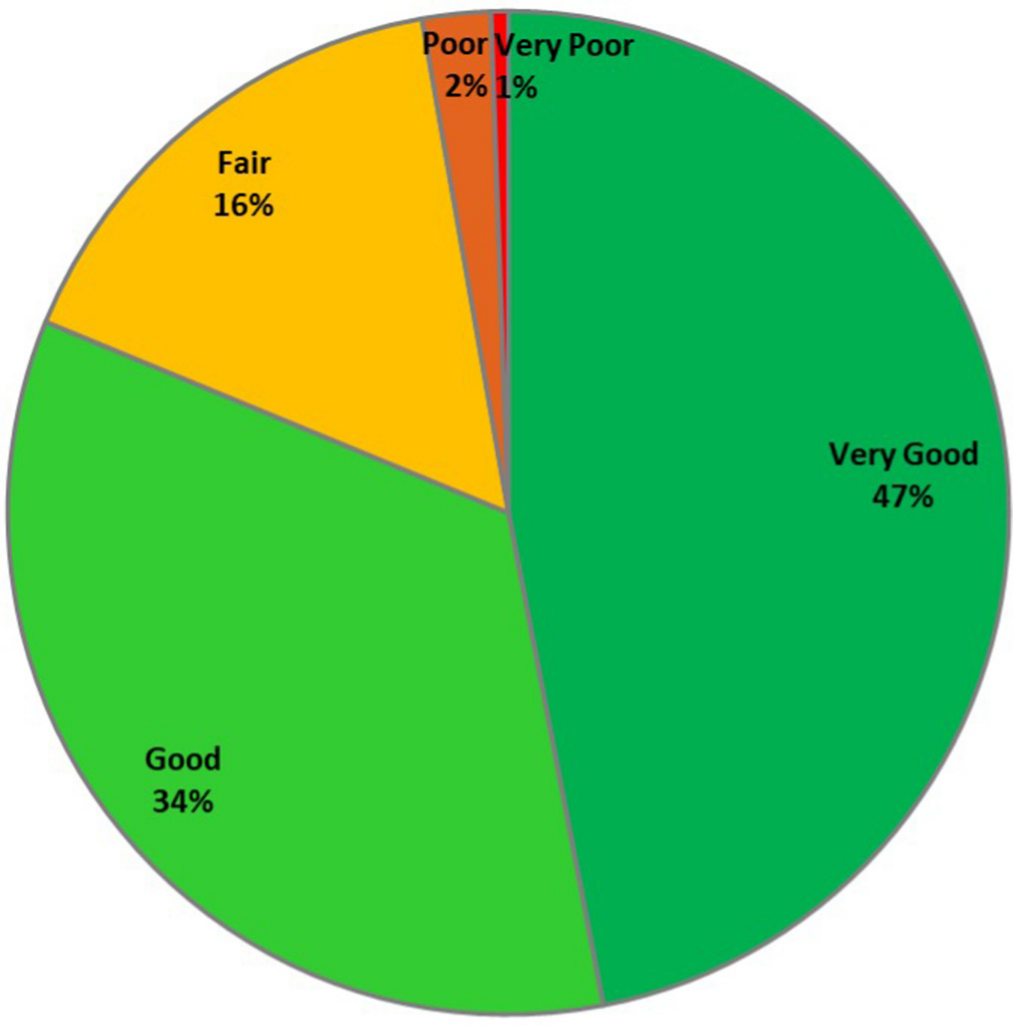
**Pie chart of the roadmap quality score classification for all Dynamic Coronary Roadmap procedures.** In the Dynamic Coronary Roadmap arm, the roadmap quality was scored by the interventional cardiologist performing the procedure on a scale of “very good,” “good,” “fair,” “poor,” or “very poor,” where both the roadmap representation of the coronary artery tree and the accuracy of the roadmap overlay location during fluoroscopy were considered (see Table 1). The roadmap quality was deemed clinically usable when rated “very good,” “good,” or “fair,” which made up 97.2% of cases.

For all the DCR-guided PCI procedures, the roadmap quality score classification is depicted in Figure 2, showing that 97% of the roadmaps were scored as clinically usable (ie, a “very good,” “good,” or “fair” rating). To allow for sufficient sample size in each analysis group, study data was split into 3 groups, ie, the “DCR good” (with a roadmap quality score of “very good” and “good”), “DCR fair/poor” (roadmap quality score “fair,” “poor,” and “very poor”) and “Control” (ie, no DCR) groups.

No significant differences in patient demographics and clinical characteristics between the 3 groups (Table 2) were found. Most of the procedural details in Table 3 do not show differences between the 3 groups; however, some statistical differences between the groups were found, ie, type of PCI (ad hoc vs planned), the first tertile of the SSv (metric for PCI complexity), intravascular ultrasound (IVUS) use, and physician experience.

**Table 2:** Baseline demographics and clinical procedural characteristics of percutaneous coronary intervention patients randomized to the DCR (split per roadmap quality score “very good/good” and “fair/poor/very poor”) and control arms.

	DCR good (very good/good) (n = 147)	DCR fair/poor (fair/poor/very poor) (n = 34)	Control (n = 184)	*P* value
Age, y	66.2 ± 11.4	65.4 ± 8.8	65.8 ± 10.8	.898
Male sex	117 (79.6)	28 (82.4)	143 (77.7)	.802
Weight, kg	84.0 ± 19.7	91.0 ± 20.8	82.9 ± 15.7	.107
Body mass index, kg/m^2^	28.6 ± 5.5	30.2 ± 6.1	28.5 ± 4.7	.285
Diabetes mellitus	50 (34.0)	15 (44.1)	72 (39.1)	.523
Hypertension (>140/90 mm Hg)	109 (74.1)	27 (79.4)	131 (71.2)	.481
Chronic kidney disease	13 (8.8)	5 (14.7)	26 (14.1)	.333
Prior revascularization	49 (33.3)	12 (35.3)	62 (33.7)	.984
Coronary artery bypass graft	10 (6.8)	2 (5.9)	16 (8.7)	.768
Prior myocardial infarction	41 (27.9)	14 (41.2)	56 (30.4)	.350
Severe left ventricular ejection fraction (<30%)	2 (1.4)	0 (0.0)	6 (3.3)	.397
eGFR, mL/min/1.73 m^2^	76.4 ± 20.3	78.3 ± 16.8	76.1 ± 19.8	.830
Permanent atrial fibrillation	12 (8.2)	2 (5.9)	18 (9.8)	.734
Chronic obstructive pulmonary disease	6 (4.1)	3 (8.8)	13 (7.1)	.431

Data given as number (%) or mean ± SD. χ^2^ test or Fisher exact test was used for categorical variables; analysis of variance or Kruskal–Wallis test was used for continuous variables.

DCR, Dynamic Coronary Roadmap; eGFR, estimated glomerular filtration rate; PCI, percutaneous coronary intervention.

**Table 3:** Procedural characteristics of PCI patients randomized to the DCR (split per roadmap quality score “very good/good” and “fair/poor/very poor”) and control arms.

	DCR good (very good/good) (n = 147)	DCR fair/poor (fair/poor/very poor) (n = 34)	Control (n = 184)	*P* value
No. of vessels treated with PCI				.235
One-vessel PCI	125 (85.0)	31 (91.2)	150 (81.5)	
Multivessel PCI	20 (13.6)	2 (5.9)	31 (16.8)	
No PCI	2 (1.4)	1 (2.9)	3 (1.6)	
Type of PCI				.017
Ad hoc PCI	82 (55.8)	27 (79.4)	119 (64.7)	
Planned PCI	63 (42.9)	6 (17.6)	62 (33.7)	
No PCI	2 (1.4)	1 (2.9)	3 (1.6)	
Treated vessel				
Left main artery	5 (3.4)	0 (0.0)	4 (2.2)	.596^a^
Left anterior descending artery	78 (53.1)	14 (42.2)	90 (48.9)	.466
Left circumflex artery	43 (29.3)	8 (23.5)	60 (32.6)	.544
Right coronary artery	35 (23.8)	13 (38.2)	50 (27.2)	.205
Lesion complexity				
Lesion length >20 mm	85 (57.8)	16 (47.1)	109 (59.2)	.433
Heavy calcification	26 (17.7)	5 (14.7)	27 (14.7)	.764
Bifurcation lesion	38 (25.9)	4 (11.8)	52 (28.3)	.133
Trifurcation lesion	2 (1.4)	0 (0.0)	1 (0.5)	.691^a^
Severe tortuosity	13 (8.8)	0 (0.0)	10 (5.4)	.132
Thrombotic lesion	6 (4.1)	0 (0.0)	6 (3.3)	.681^a^
Aorto-ostial stenosis	1 (0.7)	0 (0.0)	1 (0.5)	>.99^a^
Diffusely diseased and narrowed lesion	26 (17.7)	5 (14.7)	39 (21.2)	.587
PCI complexity score, SSv				
First tertile (low)	2.4 ± 0.6 (n = 40)	1.7 ± 1.1 (n = 16)	2.3 ± 0.7 (n = 61)	.045
Second tertile (medium)	5.5 ± 1.1 (n = 51)	5.8 ± 1.0 (n = 13)	5.7 ± 1.1 (n = 64)	.705
Third tertile (high)	10.1 ± 2.5 (n = 54)	9.3 ± 0.5 (n = 4)	9.8 ± 2.1 (n = 56)	.632
Lesions treated per procedure	1.2 ± 0.4	1.1 ± 0.2	1.2 ± 0.4	.241
Stents per procedure				.679^a^
1	94 (63.9)	23 (67.6)	116 (63.0)	
2	35 (23.8)	8 (23.5)	43 (23.4)	
3 or more	12 (8.2)	2 (5.9)	21 (11.4)	
No PCI	4 (2.7)	0 (0.0)	1 (0.5)	
Missing	2 (1.4)	1 (2.9)	3 (1.6)	
Total stent length, mm	33.2 ± 22.1	29.5 ± 15.3	34.7 ± 21.2	.409
IVUS use	69 (46.9)	5 (14.7)	61 (33.2)	<.001
Physiology assessment, iFR/FFR	15 (10.2)	1 (2.9)	11 (6.0)	.200
Physician experience, y	14.2 ± 8.0	18.7 ± 7.9	13.3 ± 7.8	<.001

Data given as number (%) or mean ± SD. χ^2^ test or Fisher exact test was used for categorical variables; Kruskal–Wallis test was used for continuous variables.

DCR, Dynamic Coronary Roadmap; FFR, fractional flow reserve; iFR, instantaneous wave-free ratio; IVUS, intravascular ultrasound; PCI, percutaneous coronary intervention; SSv, the treated vessel SYNTAX score; SYNTAX score, The Synergy Between Percutaneous Coronary Intervention with Taxus and Cardiac Surgery score.

a Fisher exact test.

**Figure 3:**
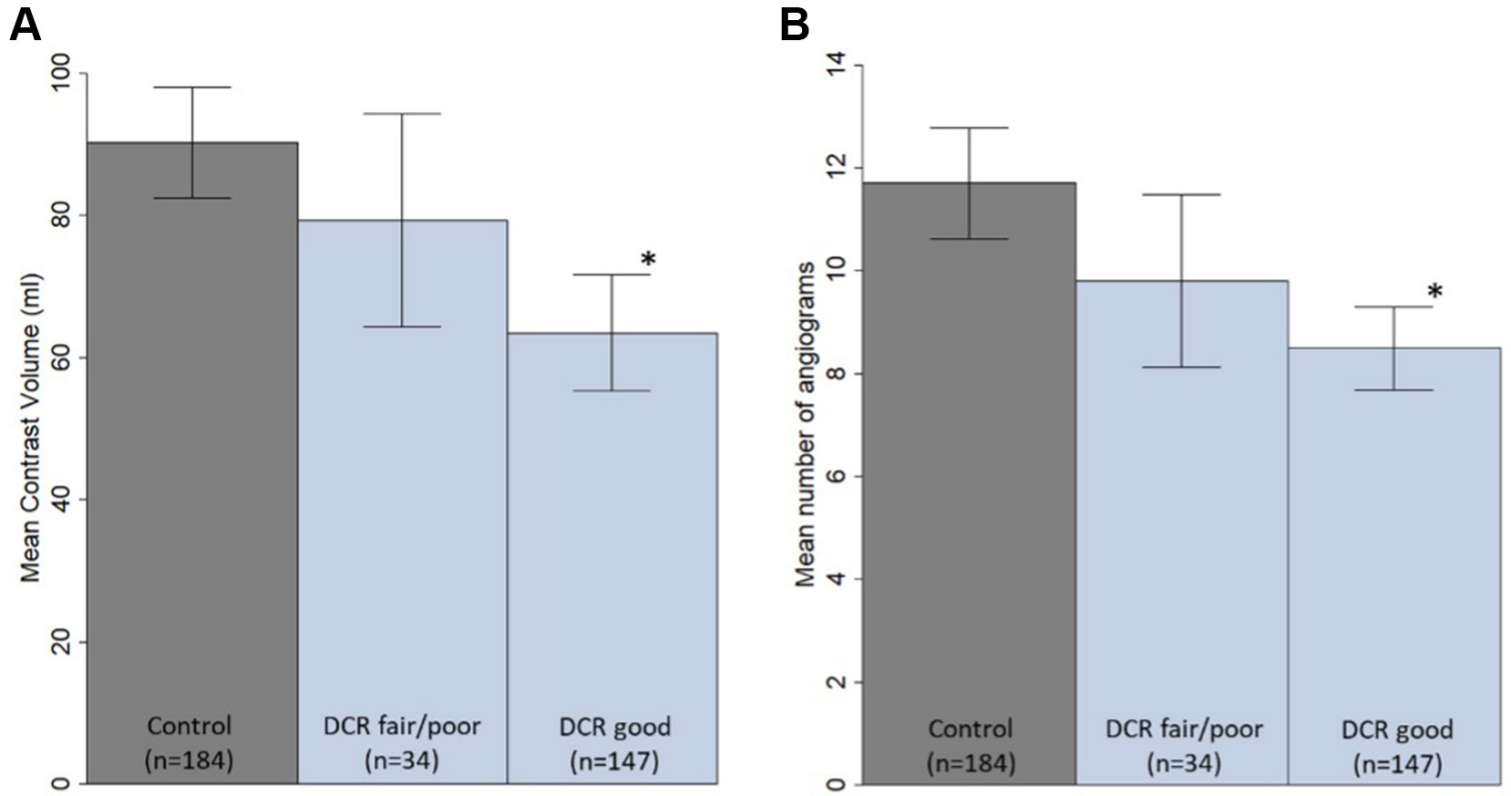
**Primary and secondary outcomes of the Dynamic Coronary Roadmap for Contrast Reduction randomized controlled trial for the “Control,” “Dynamic Coronary Roadmap (DCR) fair/poor,” and “DCR good” groups.** (**A**) Mean contrast volume used per percutaneous coronary intervention (primary objective) and (**B**) Mean number of cine angiograms obtained per percutaneous coronary intervention (secondary objective) in “Control” group, “DCR fair/poor” (with a roadmap quality scores “fair,” “poor,” and “very poor”) and the “DCR good” (roadmap quality score “very good” and “good”). The error bars represent the 95% CI of the mean and *P* values are based on a Kruskal–Wallis test with multiple comparisons according to the Dwass Steel–Critchlow–Fligner method. An asterisk (∗) is indicated next to the error bar of the DCR groups when *P* < .05 relative to the Control group.

**Supplementary Figure S2:**
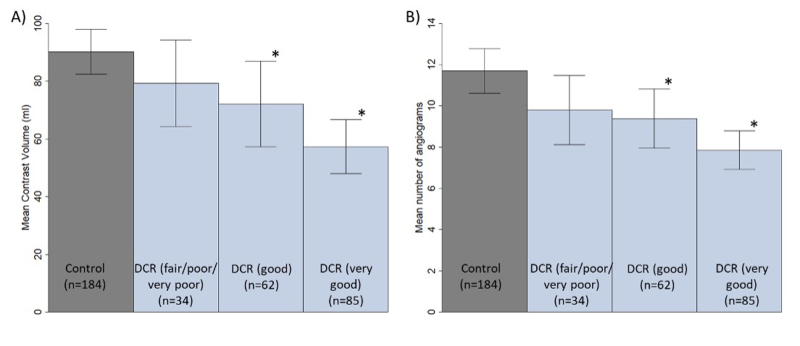
Primary and secondary outcomes of the DCR4Contast randomized control trial for the “Control”, “DCR fair/poor/very poor”, “DCR good” and “DCR very good” groups. (**A**) Mean contrast volume used per PCI (primary objective) and (**B**) Mean number of cine angiorgrams obtained per PCI (secondary objective) in the “Control”, “DCR fair/poor/very poor”, “DCR good”, and “DCR very good” groups. The error bars represent the 95% Confidence Interval of the mean and the p-value is based on log-transformed data. An asterisk (∗) is indicated next to the error bar when *P* < .05 relative to the Control group. DCR, Dynamic Coronary Roadmap; PCI, percutaneous coronary intervention.

As is shown in Figure 3 and Table 4, there was a significant reduction in the amount of contrast used per PCI under DCR guidance compared with conventional angiography-guided PCI and was lower when the roadmap was scored better: 63.5 ± 50.3 mL “DCR good” vs 79.3 ± 42.8 mL “DCR fair/poor” vs 90.2 ± 53.3 mL “Control” (*P* < .001). Similarly, the number of contrast enhanced angiograms was significantly lower in the DCR arm compared with the control arm and was again lower when the roadmap was scored better: 8.5 ± 4.9 runs “DCR good” vs 9.8 ± 4.8 runs “DCR fair/poor” vs 11.7 ± 7.4 runs “Control” (*P* < .001). Supplemental Figure S2 shows the results when the “DCR good” group is split further into “very good” and “good” roadmap quality score. The trend of lower iodinated contrast volume usage and smaller number of angiograms continued when the roadmap was scored better.

**Table 4:** Primary and secondary outcomes of the Dynamic Coronary Roadmap for Contrast Reduction trial for the DCR (split per roadmap quality score “very good/good” and “fair/poor/very poor”) and control arms.

	DCR good (very good/good) (n = 147)	DCR fair/poor (fair/poor/very poor) (n = 34)	Control (n = 184)	*P* value
Primary endpoint: iodinated contrast volume, mL				
Mean ± SD	63.5 ± 50.3	79.3 ± 42.8	90.2 ± 53.3	<.001^a^
95% CI of mean	55.2-71.7	64.3-94.2	82.4-97.9	
Median (Q1-Q3)	52.3 (32.0-79.4)	64.9 (45.0-110.0)	78.9 51.0-115.6)	
Secondary endpoint: no. of contrast enhanced cine angiographic X-ray runs				
Mean ± SD	8.5 ± 4.9	9.8 ± 4.8	11.7 ± 7.4	<.001^a^
95% CI of mean	7.7-9.3	8.1-11.5	10.6-12.8	
Median (Q1-Q3)	7 (5-10)	9 (7-13)	10 (7-14)	
PCI complexity, SSv				.054
First tertile (low; SSv < 4)	40 (27.2)	16 (47.1)	61 (33.2)	
Second tertile (medium; 4 ≤ SSv < 8)	51 (34.7)	13 (38.2)	64 (34.8)	
Third tertile (high; SSv ≥ 8)	54 (36.7)	4 (11.8)	56 (30.4)	
No PCI	2 (1.4)	1 (2.9)	3 (1.6)	
Procedural success	144 (98.0)	33 (97.1)	183 (99.5)	.216^b^
In-hospital MACCE				
Periinterventional	1 (0.7)	0 (0.0)	0 (0.0)	>.496^b^
Postinterventional	2 (1.4)	0 (0.0)	0 (0.0)	.339^b^
Acute kidney injury	4 (2.7)	0 (0.0)	5 (2.7)	>.99^b^

Data given as number (%), mean ± SD, or median (first and third quartile). χ^2^ test or Fisher exact test was used for categorical variables. Kruskal-Wallis test was used for continuous variables.

DCR, Dynamic Coronary Roadmap; MACCE, major adverse cardiovascular and cerebral events; PCI, percutaneous coronary intervention; SYNTAX score, The Synergy Between Percutaneous Coronary Intervention with Taxus and Cardiac Surgery score; SSv, the treated vessel SYNTAX score.

a Kruskal–Wallis test.

b Fisher exact test.

**Figure 4:**
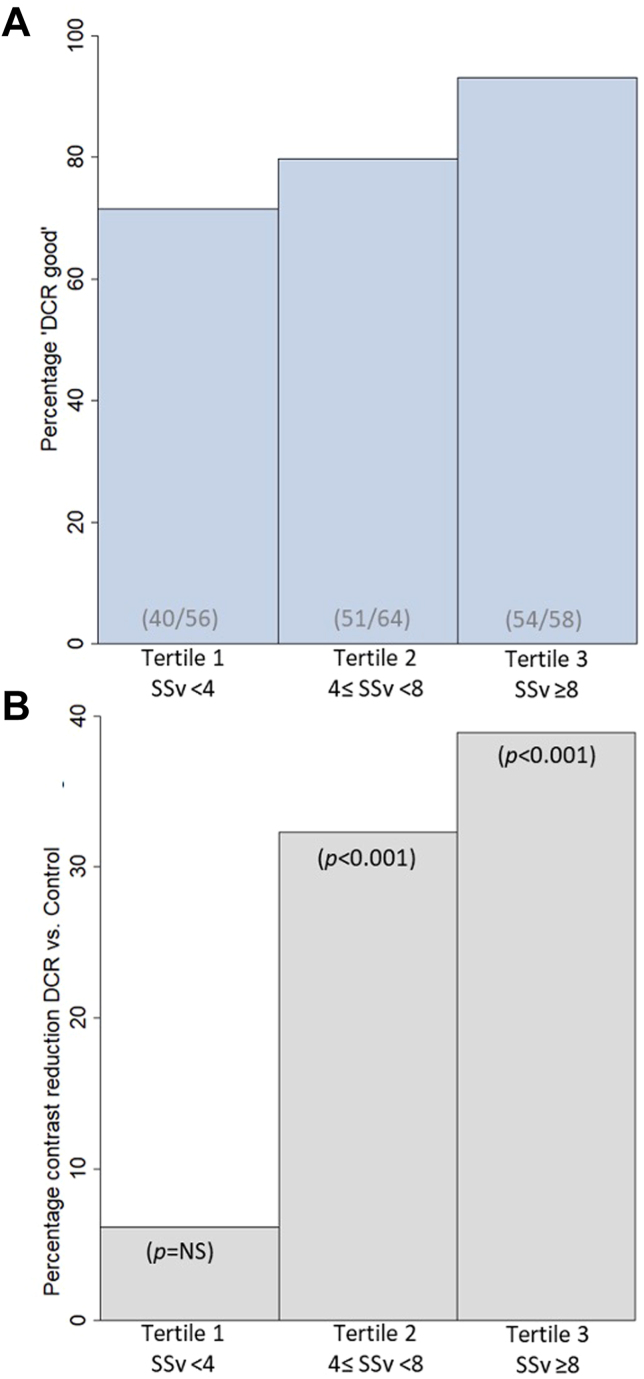
**Percentage of Dynamic Coronary Roadmap (DCR) cases in “DCR good” group and relative iodinated contrast reduction in DCR compared with standard care (Control) for increasing percutaneous coronary intervention (PCI) complexity (using the Synergy Between Percutaneous Coronary Intervention with Taxus and Cardiac Surgery (SYNTAX) score per treated vessel (SSv) as an index).** Independent of the SSv, index for PCI complexity, the percentage of DCR cases in the “DCR group” (with a roadmap quality score “very good” or “good”) is consistently high. For increasing PCI complexity, both the roadmap quality score increases as well as the percentage iodinated contrast reduction of using DCR compared with standard care (Control). (**A**) The percentage of DCR cases in the “DCR good” group (ie, roadmap quality scored “very good” or “good”) for each tertile of the SSv, an index for PCI complexity. The roadmap quality is scored better for increasing PCI complexity. The percentage “DCR good” for tertile 3 is statistically significantly different from tertile 1 (*P* = .002) and tertile 2 (*P* = .033), whereas the difference between tertile 1 and 2 is not statistically significant. (**B**) The percentage difference in iodinated contrast volume of DCR (irrespective the roadmap quality score) compared with Control for the three SSv/PCI complexity tertiles. See also Supplemental Table S1. NS, not significant

A prespecified subgroup analysis was performed based on the treated vessel SSv. The SSv was calculated for each vessel treated, ie, the number of anatomical SYNTAX score points related to the vessel being treated as an index of anatomical vessel complexity of the PCI. We then divided the patients into 3 tertiles: tertile 1, SSv < 4; tertile 2, 4 ≤ SSv < 8; and tertile 3, SSv ≥ 8, representing low, medium, and high PCI complexity, respectively. For all 3 subgroups, the percentage of cases in the “DCR good” group (with a roadmap quality score “very good” or “good”), as well as the percentage difference in iodinated contrast use when comparing all DCR with Control cases, are shown in Figure 4 and the Central Illustration (see also Table 4 and Supplemental Table S1). The roadmap quality was consistently scored “good” or “very good” across all anatomical PCI complexities using the SSv as an index. A larger proportion of roadmaps was scored “good” or “very good” for increasing PCI complexity: 40 of 56 cases in the first SSv tertile (71.4%), 51 of 64 cases in the second SSv tertile (79.7%), and 54 of 58 cases in the third SSv tertile (93.1%). With increasing PCI complexity, the relative reduction of iodinated contrast use in DCR as compared with standard care (Control) increased.

**Central Illustration:**
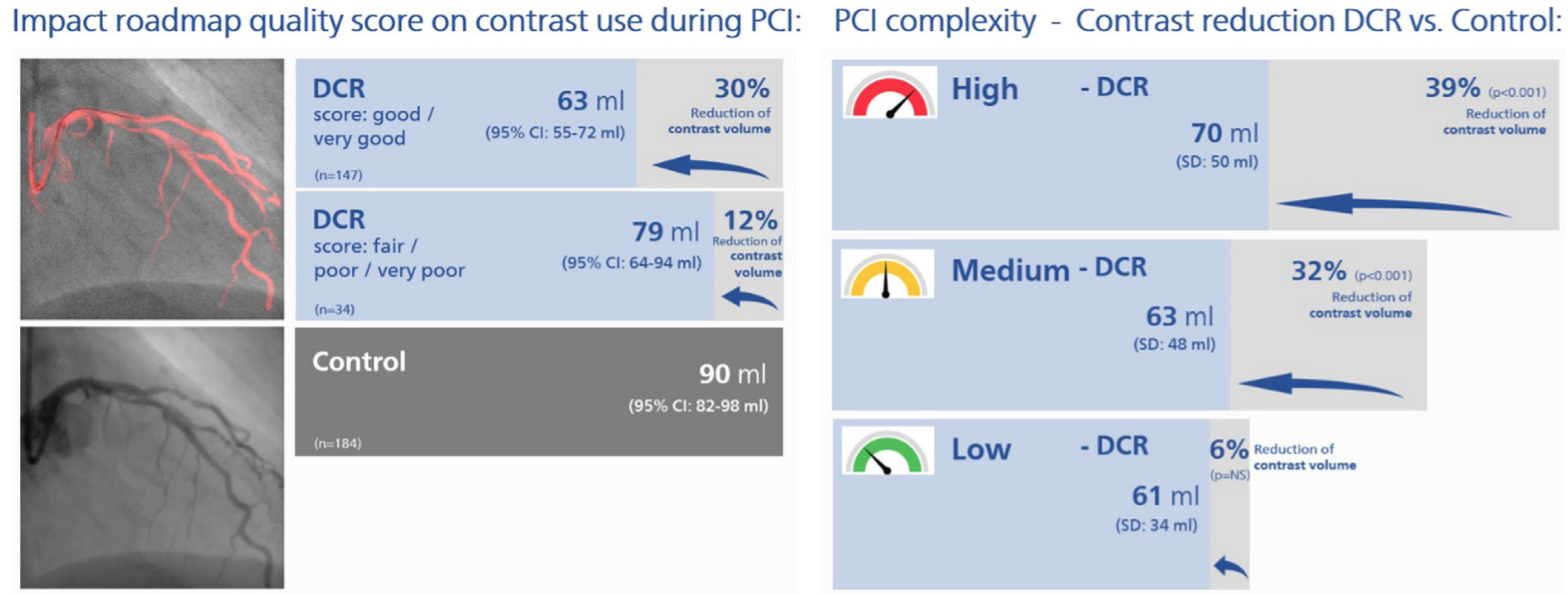
Impact of roadmap quality score on contrast used during percutaneous coronary intervention (PCI) – Contrast reduction Dynamic Coronary Roadmap (DCR) vs Control for low, medium, and high PCI complexity. NS, not significant.

The incidence of acute kidney injury or major adverse cardiovascular and cerebral events was similarly low for all groups, and there were no significant differences regarding the procedural success rates in all groups (Table 4).

## Discussion

This multicenter, international study demonstrated that the use of DCR technology during PCI procedures provides consistently high-quality roadmap support and leads to a significant reduction in the amount of iodinated contrast used.^9^ In the design phase of this trial, we expected to show that DCR reduces overall contrast volume administration by 15%, based on a previous pilot study by Yabe et al.^12^ In fact, the DCR4Contrast Trial actually demonstrated a 28% reduction when DCR was used compared with angiographic guidance alone. In addition, we did not see a statistically significant difference in the rates of IVUS and invasive physiology between groups, see also Supplemental Tables S2 and S3. This adds to the growing body of evidence supporting DCR use for contrast reduction in PCI, as shown in Table 4 and Supplemental Table S4.^9^^,^^10^

In 97% of DCR-guided procedures, the roadmap quality was scored clinically usable in terms of complete coronary artery tree representation and overlay location during fluoroscopy. This result is in line with Piayda et al,^11^ who reported sufficient roadmap quality in >98% of cases evaluated offline with good inter- and intraobserver variability. The contrast-sparing effect of the DCR system was consistent across different PCI complexities, indicating that it can potentially be applied in varied clinical settings, see also Supplemental Tables S5 and S6. Importantly, in this analysis, we found that as the case complexity increased (ie, second and third tertiles), so too did the contrast-sparing capabilities of using DCR. This is important, not only in patients at risk of chronic kidney disease, but also for complex interventions in patients with relatively normal renal function, as utilizing contrast-sparing techniques allows for more complete revascularization without having reached safe contrast limits, thus potentially increasing procedural safety. The results of this study are consistent with previous studies that have demonstrated the efficacy of the DCR technology in reducing contrast use and improving procedural outcomes.^9^^,^^12^, ^11^, ^13^, ^14^, ^15^, ^16^

**Supplementary Figure S3:**
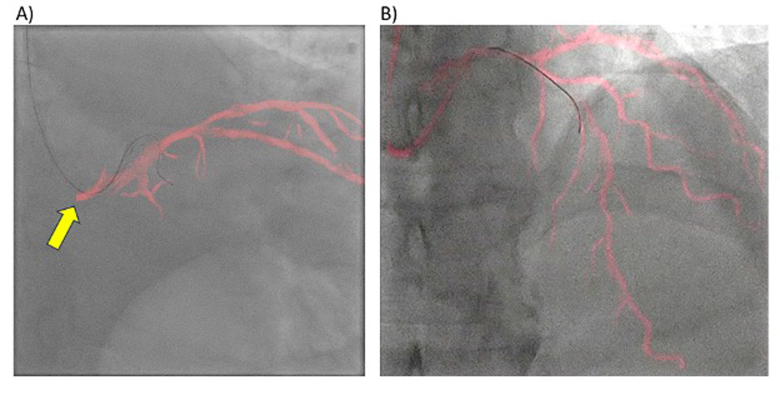
Examples of poor and good roadmap quality. Two examples of fluoro imaging with a dynamic coronary roadmap superimposed in red color, which is generated by the DCR software based on a prior coronary angiogram. (**A**) Poor roadmap quality because the guiding catheter was not sufficiently in view during the acquisition of the related coronary angiogram. This resulted in insufficient coverage of the guide catheter in the red roadmap overlay (indicated by the yellow arrow), which greatly reduced displaying the roadmap overlay in the correct location on the fluoro image by the DCR tool. (**B**) Good roadmap quality by proper visualization of the guide catheter during prior angiography, resulting in correctly displaying the roadmap overlay on fluoroscopy by the DCR technology.

A small number of simple steps can improve the roadmap quality in general. It is important to visualize the guide catheter tip properly when taking the set-up angiogram as this feature is used by the roadmapping algorithm. It is also important to ensure the guide is properly engaged to ensure proper filling of the coronary tree, ensuring that at least 3 cardiac cycles in an angiogram are captured to ensure the roadmap is created adequately. Collimation is important to ensure the image is optimized and radiation is reduced and, where possible, to exclude other cardiac devices such as pacemaker leads, etc. Adhering to all of the above typically gives high-quality roadmaps, where proper guide engagement may impact roadmap quality most. An example of good and poor roadmap quality is shown in Supplementary Figure S3. Once a roadmap has been created, it is stored automatically. If the operator was to change C-arm position, then subsequently return to the original projection in which the roadmap was created, it will automatically be redisplayed and may be reused to reduce further need for contrast.

To allow for sufficient samples in each analysis group, we added the “fair” scoring DCR cases to the “DCR fair/poor” group, although these cases were deemed clinically usable. In the procedural characteristics (Table 3), statistically significant differences were reported for the type of PCI, the first tertile of the PCI complexity using the treated vessel SYNTAX score (SSv), IVUS use, and physician experience. The higher representation of cases with these characteristics can be explained by 1 center scoring the roadmap quality significantly lower (see Supplemental Table S7). The possible dependence of physician experience on reducing contrast using DCR was recently evaluated by Quast et al^17^ in a subanalysis of the single-center randomized trial by Piayda et al.^13^ They confirmed that DCR significantly reduces usage of iodinated contrast in PCI compared with standard care without DCR (Control) irrespective of the experience level of the 2 performing investigators. Investigator A (high experience level—2800 coronary interventions) used 43.39 mL DCR vs 68.51 mL Control (*P* < .001), whereas investigator B (medium experience level—1300 coronary interventions) used 29.46 mL DCR vs 70.8 mL Control (*P* < .001). Investigator B, with medium experience level, used significantly less contrast when using DCR compared with high experience level investigator A (*P* = .047). Procedural success was 100% for both investigators, and no serious in-hospital adverse events were observed. Therefore, the DCR navigation tool is safe and effective in reducing iodinated contrast in PCI clinical routine irrespective of the experience level of the performing physician.^17^

As with any new technology, it was important to verify that medical conditions encountered in real-world practice do not impact the ability of the DCR technology to provide high-quality roadmaps and, ultimately, to fulfill its aim of reducing contrast administration during PCI. However, Table 2 shows that no statistical difference was found for patients with permanent atrial fibrillation, chronic kidney disease, prior coronary artery bypass graft, chronic obstructive pulmonary disease, or diabetes mellitus. This further supports the indication that the DCR system provides a consistently high-quality roadmap for PCI navigation guidance in a real-world patient population.

### Limitations

A cause of bias may be the fact that this study was unblinded, since DCR is a visualization tool. In addition to this, this study was not powered for clinical outcomes. Further studies are needed to confirm the long-term clinical impact of the contrast-sparing effect of DCR in PCI procedures. Another limitation of this study is that the top enrolling center, with operators with an average of 15+ years, predominantly performed ad hoc PCI in vessels without IVUS guidance and predominantly in vessels that fell into the first PCI complexity tertile (SSv < 4). Scoring of the roadmaps was left to the discretion of the investigator immediately after the procedure, which is inherently subjective despite prespecified definitions and may have therefore constituted a cause of bias.

## Conclusions

In this multicenter, international study, the use of DCR technology provided consistent high-quality roadmap support in PCI procedures and led to a significant reduction in the amount of iodinated contrast used during PCI procedures compared with conventional angiography-guided PCI. It also showed that the DCR technology delivers consistent high-quality roadmap support for PCI. The contrast-sparing effect of DCR was observed to increase as PCI complexity increased, suggesting that it has the potential to reduce the risk of CI-AKI in common clinical settings. The results of this study contribute to the growing body of evidence supporting the use of DCR as a valuable tool in the PCI toolbox. Further studies are needed to confirm the long-term clinical impact of the contrast-sparing effect of DCR.
