# Supplementary material for: Real-World Evaluation of PCI Guidance Using Dynamic Coronary Roadmap: A DCR4Contrast Trial Secondary Analysis
Source: J Soc Cardiovasc Angiogr Interv. 2025 Feb 7;4(4):102504. doi: 10.1016/j.jscai.2024.102504 (PMC12038265; doi:10.1016/j.jscai.2024.102504)
Supplement: Supplementary Tables [file mmc1.docx]

**Supplementary Material:**

**Supplementary Table 1. Primary endpoint (contrast volume) per PCI complexity as previously published**^10^**.**

| **Per PCI procedure** | **DCR**  **(n=179)** | **Control**  **(n=177)** | ***p-value*** |
| --- | --- | --- | --- |
| **Contrast volume (ml) per PCI complexity (Syntax Score per PCI treated vessel)**   - 1^st^ tertile (low) [SSv<4] - 2^nd^ tertile (medium) [4≤SSv<8] - 3^rd^ tertile (high) [SSv≥8] | 60.9 (34.1)  55.0 [35.8 – 79.0]  62.9 (47.8)  49.0 [30.0 – 75.0]  70.4 (49.6)  62.0 [34.3 – 90.0] | 64.9 (41.7)  54.5 [37.0 – 83.0]  92.9 (51.7)  78.5 [52.8 – 122.0]  115.2 (60.7)  109.4 [75.0 – 144.7] | 0.365*^#^*  <0.001*^#^*  <0.001*^#^* |

Data given as mean (standard deviation, SD) or median [1^st^ and 3^rd^ Quartile]. (^#^One-sided p-value t-test on log-transformed data). DCR: Dynamic Coronary Roadmap, PCI: Percutaneous Coronary Intervention, SSv: Syntax Score per PCI treated vessel.

**Supplementary Table 2. Contrast use in PCI with and without IVUS support**

|  | **PCI without IVUS (n=230)** | **PCI with IVUS (n=135)** | ***p-value*** |
| --- | --- | --- | --- |
| **Iodinated contrast volume, ml** | 79.6±49.5 | 76.5±57.7 | 0.220* |

Data given as mean ± standard deviation. *Wilcoxon rank-sum test. IVUS: intravascular ultrasound, PCI: percutaneous coronary intervention.

**Supplementary Table 3. Contrast use in PCI with and without IVUS support split per study arm**

|  | **DCR (n=181)** | **Control (n=184)** | ***p-value*** |
| --- | --- | --- | --- |
| **Iodinated contrast volume, ml**   - **PCI without IVUS** - **PCI with IVUS** | 71.6±49.3 (n=107)  59.1±48.6 (n=74) | 86.4±48.9 (n=123)  97.7±61.1 (n=61) | 0.006*  <0.001* |

Data given as mean ± standard deviation. *Wilcoxon rank-sum test. DCR: Dynamic Coronary Roadmap; IVUS: intravascular ultrasound, PCI: percutaneous coronary intervention.

**Supplementary Table 4. Contrast use for diagnostics in ad hoc PCI for each study arm**

| **Ad hoc PCI** | **DCR (n=111)** | **Control (n=121)** | ***p-value*** |
| --- | --- | --- | --- |
| **Iodinated contrast volume (diagnostics only), ml** | 70.9±27.5 | 75.3±33.9 | 0.458* |

Data given as mean ± standard deviation. *Wilcoxon rank-sum test. DCR: Dynamic Coronary Roadmap.

**Supplementary Table 5. Contrast use in PCI using femoral access vs. radial access**

|  | **Femoral access (n=51)** | **Radial access (n=306)** | ***p-value*** |
| --- | --- | --- | --- |
| **Iodinated contrast volume, ml** | 83.6±65.0 | 77.6±50.5 | 0.770* |

Data given as mean ± standard deviation. *Wilcoxon rank-sum test. DCR: Dynamic Coronary Roadmap.

**Supplementary Table 6. Contrast use in PCI using femoral access vs. radial access split per study arm**

|  | **DCR** | **Control** | ***p-value*** |
| --- | --- | --- | --- |
| **Iodinated contrast volume, ml**   - **Femoral access** - **Radial access** | 76.0±66.1 (n=25)  65.2±46.6 (n=152) | 90.9±64.4 (n=26)  89.8±51.3 (n=154) | 0.152*  <0.001* |

Data given as mean ± standard deviation. *Wilcoxon rank-sum test. DCR: Dynamic Coronary Roadmap.

**Supplementary Table 7. Center effect analysis for the procedural characteristics.**

|  | **Site 1**  **(n=39)** | **Site 2**  **(n=12)** | **Site 3**  **(n=95)** | **Site 4**  **(n=95)** | **Site 5**  **(n=64)** | **Site 6**  **(n=60)** | **Total**  **(n=365)** | ***p-value*** |
| --- | --- | --- | --- | --- | --- | --- | --- | --- |
| **Iodinated contrast volume, ml** | 100.7±78.0 | 77.0±39.7 | 58.5±33.9 | 91.2±46.2 | 75.1±62.1 | 79.4±47.8 | 78.4±52.7 | <0.001 |
| **Iodinated contrast volume**   - **DCR** - **Control** | 71.8±70.9  134.5±73.7 | 51.9±43.4  85.4±37.1 | 51.1±24.0  66.1±40.6 | 89.3±48.5  92.8±44.5 | 59.0±56.5  93.2±64.1 | 63.6±45.2  95.1±45.9 | 66.4±49.3  90.2±53.3 | <0.001  <0.001 |
| **Ad hoc PCI** | 6 (15.0) | 11 (91.7) | 60 (63.2) | 86 (90.5) | 33 (51.6) | 36 (60.0) | 232 (63.6) | <0.001 |
| **DCR good (very good/ good)** | 21 (100.0) | 3 (100.0) | 42 (87.5) | 22 (48.9) | 32 (94.1) | 27 (90.0) | 147 (81.2) | <0.001 |
| **DCR fair/poor (fair/poor/ very poor)** | 0 (0.0) | 0 (0.0) | 6 (12.5) | 23 (51.1) | 2 (5.9) | 3 (10.0) | 34 (18.8) | <0.001 |
| **Iodinated contrast volume**   - **DCR good** - **DCR fair/poor** | 71.8±70.9  NA | 51.9±43.4  NA | 51.7±24.2  46.8±24.6 | 82.7±55.6  95.4±41.3 | 60.1±57.8  42.5±34.7 | 65.6±47.2  45.3±10.0 | 63.5±50.3  79.3±42.8 | 0.254  0.011 |
| **PCI complexity score (SYNTAX Score per PCI treated vessel, SSv)**  **- 1^st^ tertile (low)** | 2.1±0.7  (n=7) | NA  (n=0) | 2.4±0.6  (n=38) | 1.9±1.0  (n=39) | 2.6±0.6  (n=18) | 2.6±0.5  (n=15) | 2.3±0.8  (n=117) | 0.008 |
| **IVUS use** | 19 (48.7) | 3 (25.0) | 0 (0.0) | 0 (0.0) | 55 (85.9) | 58 (96.7) | 135 (37.0) | <0.001 |
| **Physician experience (years)** | 13 [10 – 29]  (k=10) | 7 [3 – 10]  (k=5) | 11 [11 – 11]  (k=2) | 16 [11 – 27]  (k=7) | 16 [16 – 20]  (k=5) | 9 [5 – 23]  (k=5) | 11 [10 – 19]  (k=34) | <0.001 |

Data given as number (%) or mean ± standard deviation or median [1^st^ and 3^rd^ Quartile]. (Chi-Square test or Fisher Exact test for categorical variables, Kruskal-Wallis test for continuous variables). DCR: Dynamic Coronary Roadmap, IVUS: IntraVascular UltraSound, k: number of physicians per site, NA: Not Applicable, PCI: Percutaneous Coronary Intervention, SSv: SYNTAX Score per PCI treated vessel.
